# Construction of Immune-Associated Nomogram for Predicting the Recurrence Survival Risk of Stage I Cervical Cancer

**DOI:** 10.1155/2021/6699131

**Published:** 2021-07-09

**Authors:** Yajuan Wang, Lejing Zhang, Bin Wang, Yuanfang Cheng

**Affiliations:** Sanquan College of Xinxiang Medical University, West of Changjiang Avenue, Pingyuan New Area, Xinxiang City, Henan Province, China

## Abstract

**Background:**

Various studies reported that the prognosis of patients with cervical cancer (CC) was significantly associated with immunity, whereas limited studies have explored whether immune-associated genes could be classifiers for recurrence-free survival (RFS) of stage I CC. Thus, an improved immune-related gene signature for stage I CC patients' prognosis is urgently required.

**Materials and Methods:**

We retrospectively analyzed the gene expression profiles of stage I CC patients in the GSE44001 set from the Gene Expression Omnibus (GEO) database. The stage I CC patients were randomly divided into the training group and the internal validation group. The training patients were adopted to develop a prognostic immune gene-based signature; meanwhile, the internal validation patients were used to validate the power of the selected immune gene-related signature using univariate Cox proportional hazard analysis, least absolute shrinkage and selection operator (LASSO), and multivariate Cox regression analysis. The accuracy and reliability of the immune gene-related signature were evaluated based on Kaplan-Meier analysis and time-dependent receiver operating characteristic (ROC) curves.

**Results:**

High power of the 8-immune gene signature was found on the basis of ROC analysis (AUC at 1, 3, and 5 years were exhibited in the internal validation group (0.702, 0.715, and 0.728, respectively), external validation group (0.702, 0.825, and 0.842, respectively), and entire GEO dataset (0.840, 0.894, and 0.852, respectively)). Besides, *C*-index, ROC, calibration plots, and decision curve analysis (DCA) also acted well in our nomogram, suggestive of a high ability of the nomogram to elevate the prognostic prediction of stage I CC patients.

**Conclusions:**

In this study, we successfully constructed an integrated 8-immune gene-based signature which could accurately identify patients with low prognostic risk from those with high prognostic risk. In addition, we developed an immune-related nomogram which can elevate the prognostic prediction of stage I CC patients.

## 1. Introduction

Cervical cancer (CC) is the fourth most frequently diagnosed malignancy in women worldwide [1]. CC accounts for about 10% of cancer-associated deaths in women worldwide, and there are an estimated 560,000 new cases in 2018 [2, 3]. Standard therapies which include chemotherapy, radiotherapy, and surgical resection have improved the prognostic management of early-stage CC, whereas it is hard to prevent metastasis and recurrence of CC, which results in the majority of CC deaths [4, 5]. Therefore, new therapeutic methods and novel hallmarks that provide prognostic information for CC patients are urgently required.

Previous studies suggested that the immune system was a determining index during carcinoma initiation and progression [6, 7]. Accelerating evidence has showed that immune genes may serve as a hallmark of cancer. For example, Shen et al. developed and validated an immune gene set-based prognostic signature in ovarian cancer [8]. Tsakonas et al. showed that an immune gene expression signature distinguished central nervous system metastases from primary tumors in non-small-cell lung cancer [9]. Li et al. developed and validated an individualized immune prognostic signature in early-stage nonsquamous non-small-cell lung cancer [10]. Cheng et al. identified an immune-related risk signature for glioblastoma based on bioinformatic profiling [11]. Zhou et al. revealed an immune-related six-lncRNA signature to improve prognosis prediction of glioblastoma multiforme [12]. Various studies reported that the prognosis of patients with CC was significantly correlated with immunity. For instance, Yang et al. identified a prognostic immune signature for CC to predict survival and response to immune checkpoint inhibitors [13]. Huang et al. revealed the prognostic value of the preoperative systemic immune-inflammation index in patients with CC [14]. Wang et al. showed the prognostic landscape of tumor-infiltrating immune cells in CC [15]. Chen et al. revealed the correlation between subsets of tumor-infiltrating immune cells and risk stratification in patients with cervical cancer [16]. Cui et al. identified the TCR repertoire as a novel indicator for immune monitoring and prognosis assessment of patients with CC [17], whereas limited studies have explored whether immune-associated genes could be predictors for the prognosis of stage I CC. Therefore, an improved immune-associated gene signature for stage I CC patients' prognosis is urgently needed.

In this study, we investigated the gene expression data and related clinical information of stage I CC patients in GSE44001 from the GEO database to develop an individualized prognostic signature for stage I CC patients based on bioinformatic methods. Then, the power of the 8-immune gene signature was validated via ROC analysis and Kaplan-Meier analysis. We developed a nomogram on the basis of the risk score, cancer status, and race to improve the predicted value of the 8-immune gene signature. The outcomes indicated that our nomogram was an accurate prognostic prediction tool.

## 2. Materials and Methods

### 2.1. Data Acquisition

We retrospectively analyzed the gene expression profiles of stage I CC samples in the GSE44001 set of the GEO database via the GEOquery package [18]. Only samples with both survival information and expression data available were included in the present study. In addition, genes with a lack of expression values or samples with a lack of prognostic information for recurrence were removed. Following this, immune gene data were downloaded from the ImmPort database [19]. Consequently, 1811 immune genes and 258 stage I CC cases were included to develop the immune gene-based prognostic classifier for stage I CC. Besides, 153 samples with stage I CC from the TCGA database were searched across the TCGAbiolinks package [20] which was employed as an external validation group. The detailed information of enrolled samples in GEO and TCGA databases was presented in Table S1. In addition, our article was about data mining analysis through TCGA and GEO database and not associated with ethical approval.

### 2.2. Development and Validation of the Immune-Related Signature for Stage I CC

We first performed univariable Cox regression analysis using the collected immune gene to identify the immune genes which generated a close (*P* < 0.05) correlation with RFS of stage I CC patients. Following that, the identified immune genes were adopted for LASSO analysis to determine stage I CC patients' RFS-related candidate immune genes (*P* < 0.05). Finally, we implemented multivariate Cox regression analysis according to the candidate immune genes to select RFS signature genes for stage I CC patients, and an 8-metabolic gene signature was identified as a prognostic predictor.

The stage I CC samples in the entire GEO dataset were randomly assigned into the training and internal validation groups with a ratio of 7 : 3. The training patients were adopted to build a prognostic immune gene-related signature; meanwhile, the internal validation patients were exploited to validate the reliability of the selected immune gene-related signature. After that, we established a formula by using the 8-immune gene-based signature to calculate the immune risk score for each patient. Patients were then separated into high- and low-risk cohorts based on the median cutoff of the risk score. We assessed the prognostic performance of the immune signature by comparing the sensitivity and specificity of ROC curves via R (version 4.0.0), and the AUC value was employed as an evaluation indicator. The comparison of RFS between the high-risk and low-risk cohorts was assessed by the Kaplan-Meier curve through R (version 4.0.0).

### 2.3. Quantitative Reverse Transcription-Polymerase Chain Reaction (qRT-PCR)

Total RNA was extracted from stage I CC tissues via TRIzol (Invitrogen) according to the manufacturer's instructions. The complementary DNA (cDNA) was synthesized through the reverse transcription kit (Takara, Japan) in accordance with the instructions of the manufacturer, and qRT-PCR was conducted by a stem-loop RT primer and the PrimeScript RT reagent kit (Takara Bio, Inc.) following the manufacturer's instructions. Gene expression was normalized to GAPDH and was calculated via the 2−*ΔΔ*Cq method. Each experiment was performed three times. All primer sequences are listed in Table S2.

### 2.4. Single Sample Gene Set Enrichment Analysis (ssGSEA)

ssGSEA was carried out based on the TCGA-CESC mRNA dataset across the GSVA package [21] to find the immune gene signature-based signaling pathways. We investigated the top 20 key immune gene-related pathways that had a positive association with the risk score. The stage I CC patients in the entire GEO dataset were randomly divided into the training and internal validation groups with a ratio of 7 : 3. *p* values of <0.05 were considered statistically significant.

### 2.5. Identification of the Nomogram

We implemented univariate and multivariate Cox proportional hazard analysis on the basis of the risk score and several clinic-related factors. Cox proportional hazard models were utilized for calculating hazard ratios (HR) and corresponding 95% confidence interval (CI). To elevate the predictive reliability of the 8-immune gene-based signature for stage I CC patients' RFS, a nomogram was established on the basis of the risk score, cancer status, and race via the “rms” R package. The predictive capability of our nomogram for stage I CC patients' RFS was assessed according to *C*-index, ROC, calibration plots, and DCA. The predicted results of the nomogram were illustrated in the calibrate curve, and the 45° line stood for the ideal prediction.

## 3. Results

### 3.1. Patient Characteristics

**Figure 1:**
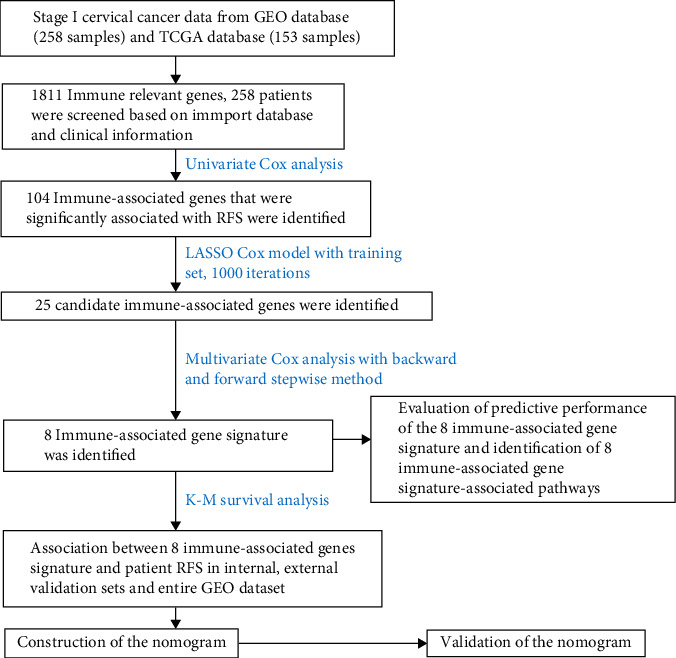
Flow chart of the present research.

A cohort containing 258 stage I CC patients with available expression data and related clinical information in the GEO database was analyzed. The clinicopathological features of the analyzed samples are showed in Table 1. A flowchart which manifested the entire process of the study is showed in Figure 1.

### 3.2. Construction of Immune-Associated Risk Signature

**Figure 2:**
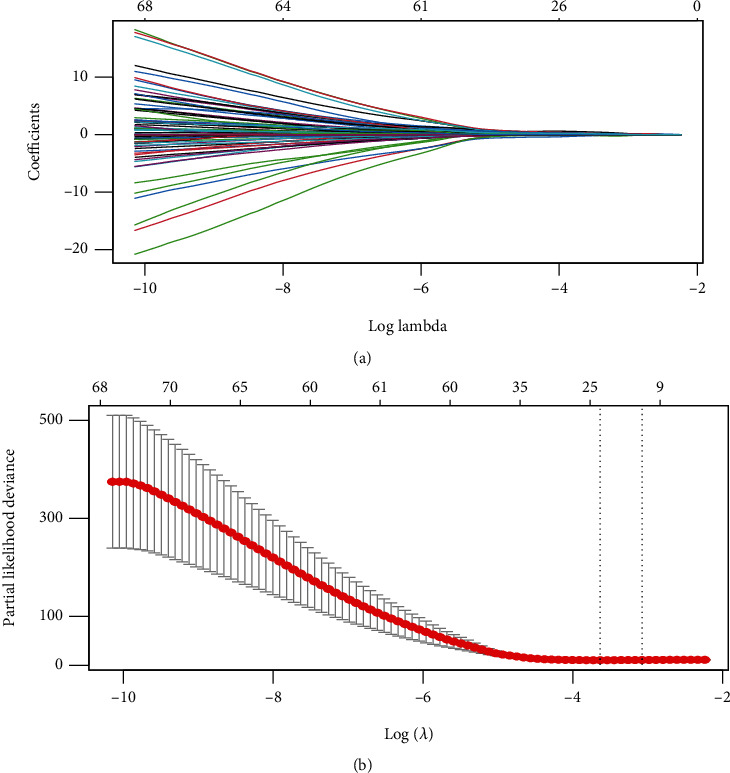
Candidate immune gene selection according to the LASSO Cox regression model. (a) 10-fold cross-validation for tuning parameter selection in the LASSO model by minimum criteria (the 1-SE criteria). (b) LASSO coefficient profiles of the 104 immune genes. A coefficient profile plot was generated against log (lambda) sequence. Vertical line was drawn at the value selected with 10-fold cross-validation, where optimal lambda resulted in 25 nonzero coefficients.

**Figure 3:**
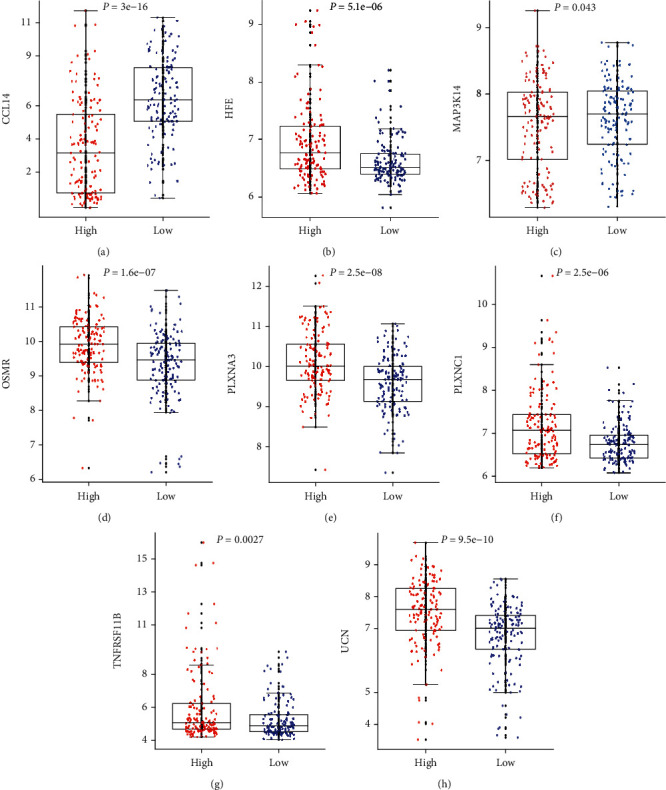
Boxplots of the 8 immune gene expression values against risk cluster in the GEO dataset. “High” and “low” referred to the high-risk and low-risk clusters, respectively. The differences between the 2 clusters were tested by the Mann-Whitney *U* test, and *p* values were exhibited in the graphs.

Univariate and LASSO Cox regression analyses were adopted to examine the association between the 2059 immune-related genes and RFS of the stage I CC patients (Table S3). The result showed that 25 immune-related genes were discovered to be importantly involved in stage I CC patients' RFS in the light of LASSO Cox regression analysis (Figures 2(a) and 2(b)). At last, 8 immune-correlated genes (CCL14, MAP3K14, HFE, UCN, TNFRSF11B, OSMR, PLXNA3, and PLXNC1) were manifested to be closely correlated with RFS of stage I CC patients on the basis of multivariate Cox analysis. Risk score = −0.151∗CCL14 + 0.769∗HFE − 0.800∗MAP3K14 + 0.672∗OSMR + 0.895∗PLXNA3 + 0.509∗PLXNC1 + 0.251∗TNFRSF11B + 0.396 ∗ UCN. The 8-immune-correlated gene signature was exploited as an indicator for RFS of stage I CC patients.  Figure 3 and Figure S1 illustrate that the high immune-correlated gene expression of HFE, OSMR, PLXNA3, PLXNC1, TNFRSF11B, and UCN had a distinctly miserable survival; nevertheless, the low immune-associated gene expression of CCL14 and MAP3K14 produced an evidently gloomy survival. In addition, 28 immune checkpoints and tumor mutation burden (TMB) from the TCGA database were also analyzed between the two risk groups (Figure S2). The result showed that the expression levels of CD4, CXCR4, LGALS9, TNFRSF4, and TNFSF4 were elevated in the high-risk group, while the expression levels of CD48, CD83, and KLRG1 were raised in the low-risk group. Importantly, the expression of TMB was raised in the low-risk group (Figure S3). Otherwise, the expression of the immune genes in CC tissues was analyzed by real-time quantitative reverse transcription-polymerase chain reaction (qRT-PCR) and immunohistochemical (IHC) assay (Figure S4). The result exhibited that the expression levels of CCL14 were reduced in CC tissues; meanwhile, the expression levels of HFE, TNFRSF11B, OSMR, and PLXNA3 were increased in CC tissues.

### 3.3. Relationship between the Immune-Associated Signature and Stage I CC Patients' RFS

**Figure 4:**
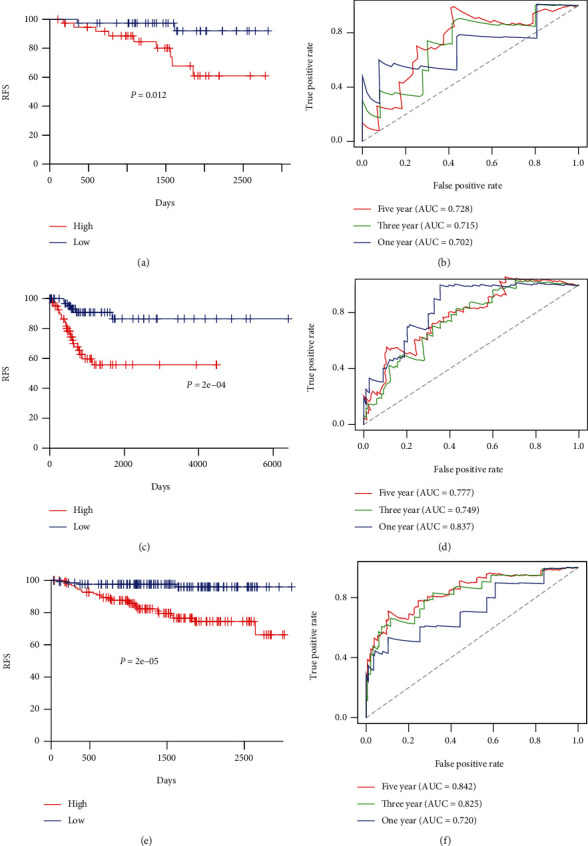
Kaplan-Meier and ROC analysis of patients with stage I CC in the internal validation, external validation, and entire GEO set, respectively. (a, c, e) Kaplan-Meier analysis with a two-sided log-rank test was implemented to test the differences in RFS between the low-risk and high-risk samples. (b, d, f) 1-, 3-, and 5-year ROC curves of the 8-immune gene signature were adopted to examine the power in predicting stage I CC patients' RFS.

The stage I CC samples were separated into the high- and low-risk groups according to the median cutoff of the risk score. Kaplan-Meier analysis was adopted to explore the difference in RFS between two groups. Survival analysis showed that the cohorts with lower risk scores had longer RFS than the high-risk cohorts, which was illustrated in the internal validation group (*p* = 0.012) (Figure 4(a)). A similar outcome was implied in the external validation group (*p* = 2*e* − 04) (Figure 4(c)) and the entire GEO group (*p* = 2*e* − 05) (Figure 4(e)).

### 3.4. Evaluation of the Predicted Capacity of the 8-Immune Gene-Correlated Signature Based on ROC Analysis

Time-dependent ROC curves were employed to test the robustness of the 8-immune-correlated gene signature. The AUC at 1, 3, and 5 years in the internal testing group were 0.702, 0.715, and 0.728, respectively (Figure 4(b)), the external testing group (0.837, 0.749, and 0.777, respectively) (Figure 4(d)), and the entire GEO group (0.702, 0.825, and 0.842, respectively) (Figure 4(f)). These results showed that the 8-immune gene signature was a firm prognostic tool.

**Figure 5:**
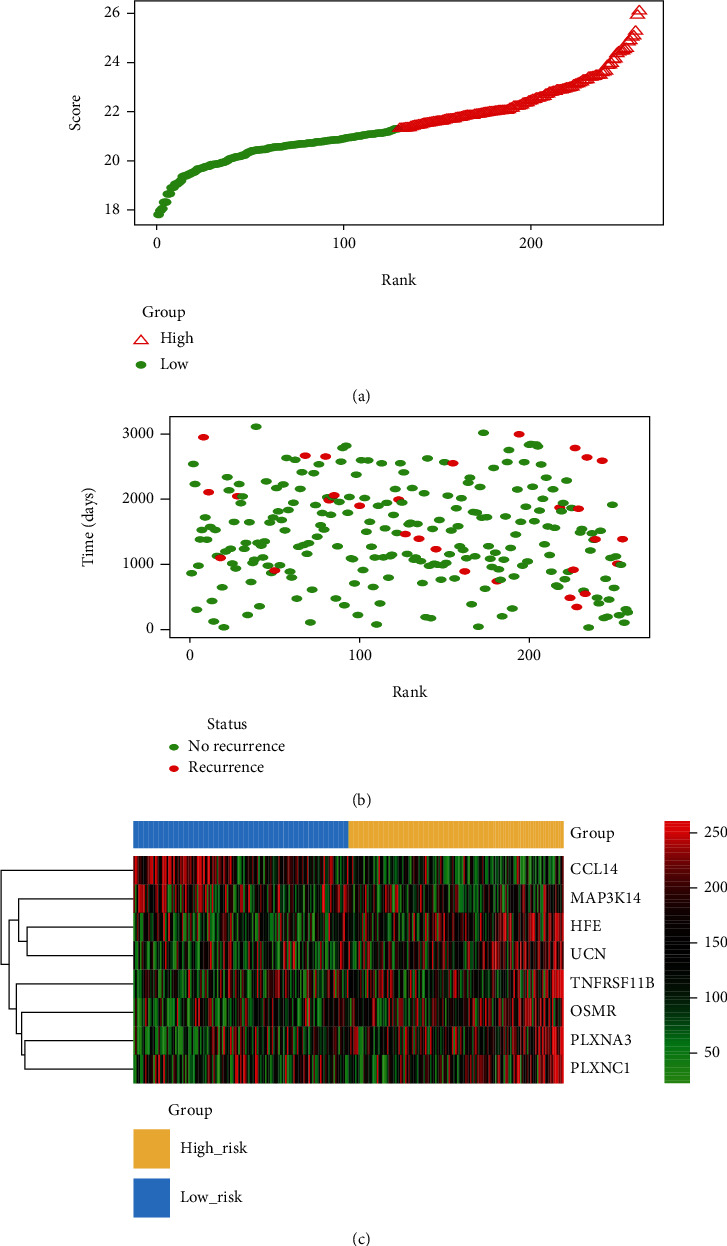
Immune gene risk score analysis of 258 stage I CC samples in the GEO dataset. (a) Metabolic gene risk score distribution against the rank of risk score. Median risk score was adopted as the cutoff point. (b) Recurrence-free survival status of stage I CC patients. (c) Heatmap of 8 immune gene expression profiles of stage I CC patients.

Following this, we ranked the immune risk scores of the stage I CC patients in the training and internal testing cohorts and analyzed their distribution (Figure 5(a)), and the survival status of stage I CC cases in the training and internal testing cohorts was illustrated on the dot plot (Figure 5(b)). We found that the groups with lower risk scores had longer RFS than the high-risk groups. Heatmap distribution of the 8 immune-correlated genes clustered through the immune risk score is exhibited in Figure 5(c), which was concordant with the result in Figure 3. A similar outcome was found in the external validation group (Figure S5), which supported the result in Figure 3.

### 3.5. Exploration of the 8-Immune Gene Signature-Correlated Biological Pathways

**Figure 6:**
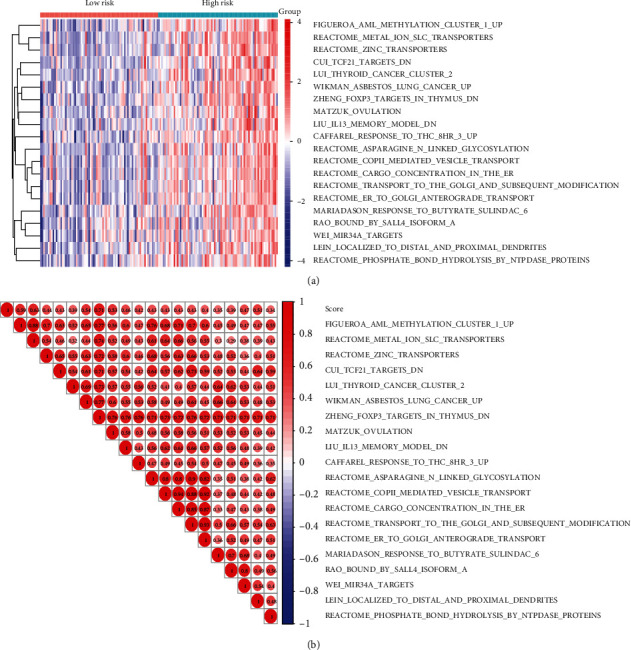
Identification of the 8-immune gene signature-associated biological pathways. (a) Heatmap of top 20 enriched pathways associated with the high-risk group. (b) Correlation graph between risk scores and top 20 pathways.

The stage I CC samples were divided into the high- and low-risk groups according to the median cutoff of the risk score. The top 20 core immune gene-activated pathways that produced a positive association with the immune risk score are illustrated in Figure 6(a) (Table S4). A significantly positive association between the enriched pathways and immune risk score is further exhibited in Figure 6(b).

### 3.6. Nomogram Construction

**Figure 7:**
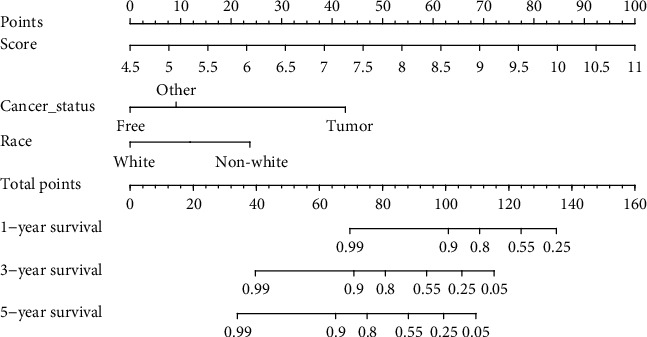
Immune gene-correlated nomogram for the prediction of RFS with stage I CC patients. The nomogram was built in the entire GEO database, with the immune gene risk score, race, and cancer status.

**Figure 8:**
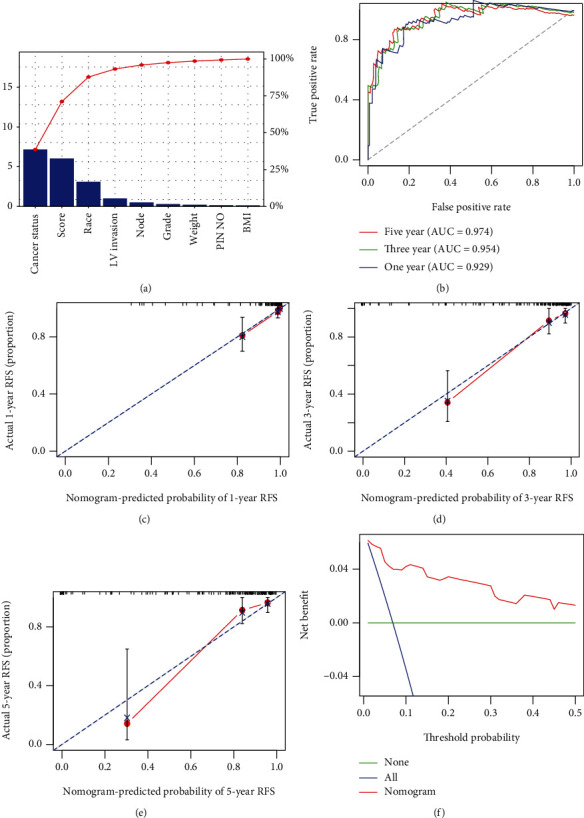
Validation of immune gene-correlated nomogram in entire GEO dataset. (a) The higher the bar chart, the larger the percentage. (b) 1-, 3-, and 5-year ROC curves for the immune gene-correlated nomogram. (c–e) Stood for the 1-, 3-, and 5-year nomogram calibration curves, respectively. The closer the dotted line fit to the perfect line, the better the predictive power of the nomogram. (f) The decision curve analysis (DCA) for the nomogram. The net benefit was plotted versus the threshold probability. The red line stood for the nomogram. The blue line stood for the treat all, and the green line stood for the treat none.

Univariate and multivariate Cox regression analyses were carried out to further analyze for independency of the 8-immune gene signature to predict stage I CC patients' RFS based on the risk score and other known clinical factors. The results indicated that the 8-immune gene signature was independent predictive factors, with a hazard ratio (HR) of 4.68 (95% CI: 2.67-8.20, *p* = 6.94*e* − 08) (Table 2). To elevate predicted robustness of the 8-immune gene-based signature for stage I CC patients' RFS across a quantitative method, we built a nomogram on the basis of the risk score, cancer status, and race (Figure 7) to predict 1-, 3-, and 5-year stage I CC patients' RFS. The importance between the risk score and the clinic-related variables is depicted in Figure 8(a). The result exhibited that *C*-index (0.911, 95% CI: 0.876-0.936), AUC (0.929, 0.954, and 0.974) (Figure 8(b)), and calibration plot illustrated that the nomogram served as a perfect predictive model (Figures 8(c)–8(e)). Besides, the DCA manifested that the nomogram produced a crucial clinical application for prognosis prediction of stage I CC patients than that in the treat all or treat none cluster. Net benefit was verified for stage I CC patients' 3-year recurrent risks (Figure 8(f)), illustrating the good capacity of our tool.

## 4. Discussion

We constructed and validated an immune gene-based signature for CC using GEO and TCGA dataset. The signature consisted of 8 immune genes with prognostic ability. A combination of the 8 immune genes (CCL14, MAP3K14, HFE, UCN, TNFRSF11B, OSMR, PLXNA3, and PLXNC1) was used as a predictor for stage I CC patients' RFS. Among the 8 immune genes, 6 immune genes (HFE, OSMR, PLXNA3, PLXNC1, TNFRSF11B, and UCN) were correlated with high risk and 2 immune genes (CCL14 and MAP3K14) were protective factors. Of note, previous researches have manifested that these above 8 immune genes were involved in carcinoma, respectively. For instance, Gu et al. suggested that CCL14 was a prognostic biomarker and correlated with immune infiltrates in hepatocellular carcinoma [22]. Mak et al. reported that the apoptosis repressor with caspase recruitment domain modulated second mitochondrial-derived activator of caspase mimetic-induced cell death via BIRC2/MAP3K14 signaling in acute myeloid leukemia [23]. Liu et al. indicated that C282Y polymorphism in the HFE gene was associated with the risk of breast cancer [24]. Shao et al. suggested that 7-hydroxystaurosporine (UCN-01) induced apoptosis in human colon carcinoma and leukemia cells independently of p53 [25]. Luan et al. found that TNFRSF11B activated Wnt/*β*-catenin signaling and promoted gastric cancer progression [26]. Hibi et al. indicated that methylation of the OSMR gene was frequently observed in noninvasive colorectal cancer [27]. Gabrovska et al. suggested that PLXNA3 may have some form of a growth-suppressive role in breast cancer [28]. Chen et al. manifested that PLXNC1 enhanced carcinogenesis through transcriptional activation of IL6ST in gastric cancer [29]. We speculated that the above 8 immune genes were also related to stage I CC.

Various studies manifested that nomograms may elevate prognostic prediction for tumors on the basis of several clinical variables via a quantitative method. For example, Chen et al. reported the transcription factor profiling to predict recurrence-free survival in breast cancer: development and validation of a nomogram to optimize clinical management [30]. Xiong et al. suggested that nomogram integrating genomics with clinicopathologic features improved prognosis prediction for colorectal cancer [31], whereas fewer studies developed a nomogram to predict the prognosis of stage I CC patients. Our nomogram was built according to clinical variables to predict the prognosis of stage I CC patients in a quantitative method; in other words, our model can predict specific survival percentages of stage I CC patients, which may elevate prognostic prediction for stage I CC patients.

Considerable researches demonstrated that the LASSO Cox regression model can be adopted to select prognostic predictors of various cancers, For example, Lan et al. identified prognostic factors and constructed a prognostic miRNA signature based on univariate Cox regression analysis and LASSO [32]. Luo et al. developed a three-miRNA signature as a novel potential prognostic hallmark in patients with clear cell renal cell carcinoma [33]. The LASSO method can minimize the log partial likelihood subject to the sum of the absolute values of the parameters which are bounded by a constant. Thus, it shrinks coefficients and generates some coefficients which are precisely zero. Consequently, it can reduce the assessment variance when providing an explicable final tool [34]. We adopted the LASSO method to select the candidate immune-associated genes significantly related to RFS of stage I CC patients for eliminating the interference of the possible multicollinearity.

Therefore, the application of LASSO analysis can elevate the prognostic prediction for RFS of stage I CC patients.

Although the 8-immune gene-based signature appears to be a potential prognostic predictor in clinical application, there are also some limitations. Firstly, the IHC results for PLXNA3 and PLXNC1 were not available. Secondly, the sample size in our external validation set was not large enough. The third limitation was that the prognostic value of the 8-immune gene-based signature was tested only by online databases, and more prospective studies should be further performed. Fourthly, we developed the nomogram according to retrospective data from the TCGA database, which may produce a hazard of selection bias.

## 5. Conclusion

We constructed an integrated 8-immune gene-based signature that was significantly related to RFS of stage I CC patients which could accurately identify patients with low prognostic risk from those with high prognostic risk. Furthermore, we evaluated the accuracy and reliability of the above signature based on Kaplan-Meier analysis and ROC curves. These results suggested that the 8-immune gene-based signature could potentially serve as a prognostic tool in stage I CC. The indicators indicated that our nomogram can elevate the prognostic prediction of stage I CC patients.

## Figures and Tables

**Table 1:** Clinical features of contained samples.

Characteristics	Training dataset (n = 181)	Testing dataset (n = 77)	External validation test (n = 153)
Stage			
IA	9 (0.05)	4 (0.052)	6 (0.039)
IB	172 (0.95)	73 (0.948)	147 (0.961)
Diameter			
≥3.0 mm	89 (0.492)	38 (0.494)	
<3.0 mm	92 (0.508)	39 (0.506)	
Grade			
G1			11 (0.072)
G2			69 (0.451)
G3			61 (0.399)
Not available			12 (0.079)
Height			
>160 cm			65 (0.425)
≤160 cm			65 (0.425)
Not available			23 (0.15)
Weight			
>70 kg			76 (0.497)
≤70 kg			64 (0.418)
Not available			13 (0.085)
BMI			
≥27			64 (0.418)
<27			64 (0.418)
Not available			25 (0.163)

**Table 2:** Univariate Cox regression analysis and multivariate Cox regression analysis outcomes based on immune gene risk score and other clinic-relevant factors.

	Univariate analysis				Multivariate analysis			
id	HR	HR.95 L	HR.95 H	*p* value	HR	HR.95 L	HR.95 H	*p* value
Cancer status	4.339719	2.851935	6.603643	7.25*e* − 12	4.680186	2.670895	8.201051	6.94*e* − 08
Score	2.951403	1.846756	4.716801	6.06*e* − 06	3.21561	1.791249	5.77259	9.13*e* − 05
Number of positive lymph nodes	1.144813	1.053136	1.244472	0.001495	0.978141	0.867004	1.103524	0.719476
Lymphovascular invasion indicator	2.093224	1.290804	3.394462	0.002746	1.80633	0.897758	3.634418	0.097396
N	1.515486	0.999303	2.298299	0.050385	0.728134	0.393761	1.346447	0.311754
Grade	1.39709	0.975019	2.001868	0.068438	1.161459	0.747212	1.805361	0.50599
Race	1.378743	0.919578	2.067178	0.120127	2.652522	1.498592	4.694991	0.000812
BMI	0.94959	0.888731	1.014617	0.125877	1.016788	0.911553	1.134172	0.765189
Weight	0.984041	0.961687	1.006915	0.170005	0.992311	0.96137	1.024249	0.63297
M	0.683123	0.326188	1.43064	0.312295				
Height	1.013923	0.959142	1.071833	0.625605				
T	1.157597	0.604218	2.217795	0.65909				
Total number of pregnancies	1.029369	0.87153	1.215795	0.733222				
Age	0.99709	0.96979	1.025158	0.836982				
Menopause status	1.019268	0.777162	1.336796	0.890296				
Keratinizing squamous cell carcinoma present indicator	0.972696	0.597031	1.584735	0.911485				
Ethnicity	0.972632	0.534771	1.769003	0.927551				
Number of successful pregnancies	1.00326	0.801568	1.255701	0.977329				
Tobacco smoking history	0.998008	0.715417	1.392223	0.990635				

## Data Availability

The gene expression and clinical data of stage I cervical cancer patients in our study were obtained from TCGA (http://tumorsurvival.org/) and GEO (https://www.ncbi.nlm.nih.gov/geo).

## Supplementary Materials

Supplementary Materials Table S1: the detailed clinical information of the samples enrolled in the present study. Table S2: all primer sequences used in this study. Table S3: hazard ratios and 95% CIs and *p* values of 1811 immune genes via univariate Cox regression analysis. Table S4: the enriched pathways related to the immune risk score. Figure S1: boxplots of 8 immune gene expression values against the risk group in the TCGA dataset. Figure S2: 28 immune checkpoints from the TCGA database were explored between the two risk groups. Figure S3: tumor mutation burden (TMB) from the TCGA database was explored between the two risk groups. Figure S4: the expression of the immune genes in CC tissues was assessed by qRT-PCR and IHC analysis. Figure S5: immune gene risk score analysis of 153 stage I CC samples in the TCGA database. (A) Metabolic gene risk score distribution against the rank of risk score. Median risk score was adopted as the cutoff point. (B) Recurrence-free survival status of stage I CC patients. (C) Heatmap of 8 immune gene expression profiles of stage I CC patients.
