# Supplementary figures and images for: Construction of Immune-Associated Nomogram for Predicting the Recurrence Survival Risk of Stage I Cervical Cancer
Source: Biomed Res Int. 2021 Jul 9;2021:6699131. doi: 10.1155/2021/6699131 (PMC8289578; doi:10.1155/2021/6699131)

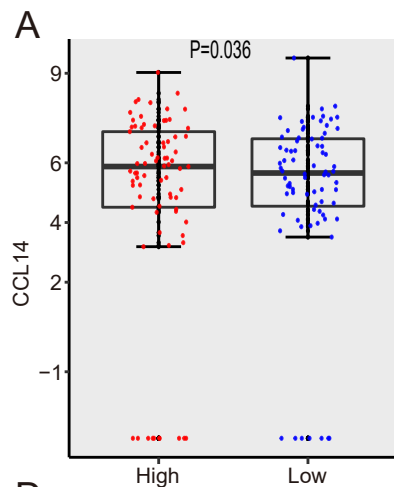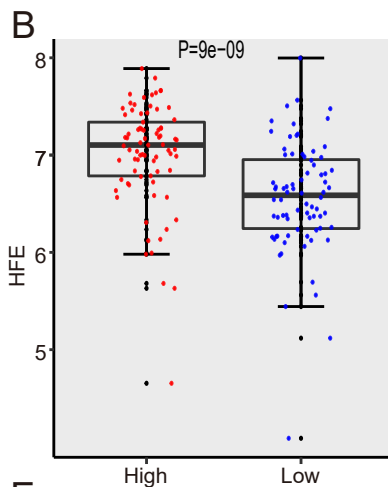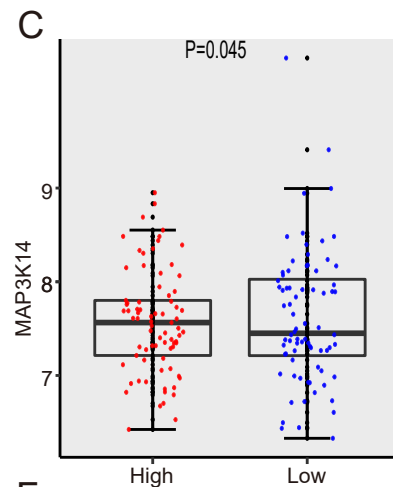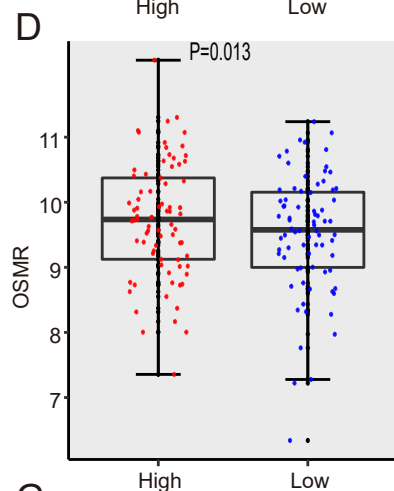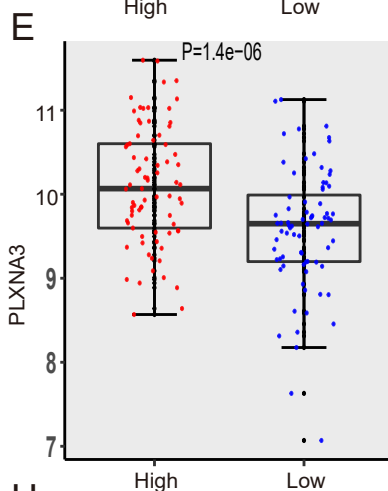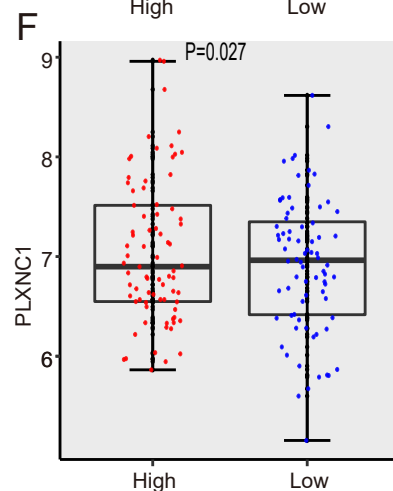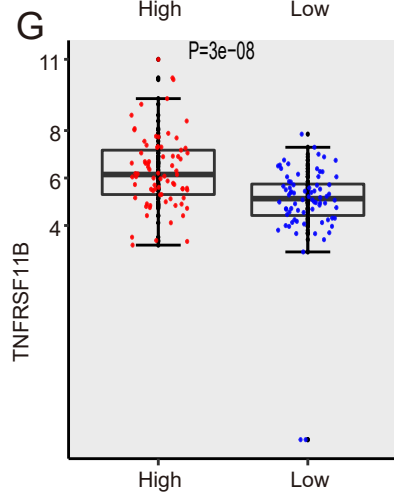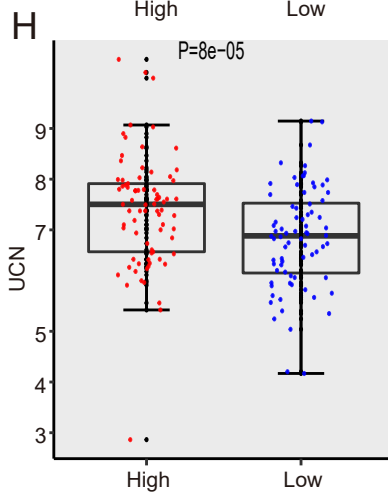

Supplement: Supplementary Materials — Table S1: the detailed clinical information of the samples enrolled in the present study. Table S2: all primer sequences used in this study. Table S3: hazard ratios and 95% CIs and p values of 1811 immune genes via univariate Cox regression analysis. Table S4: the enriched pathways related to the immune risk score. Figure S1: boxplots of 8 immune gene expression values against the risk group in the TCGA dataset. Figure S2: 28 immune checkpoints from the TCGA database were explored between the two risk groups. Figure S3: tumor mutation burden (TMB) from the TCGA database was explored between the two risk groups. Figure S4: the expression of the immune genes in CC tissues was assessed by qRT-PCR and IHC analysis. Figure S5: immune gene risk score analysis of 153 stage I CC samples in the TCGA database. (A) Metabolic gene risk score distribution against the rank of risk score. Median risk score was adopted as the cutoff point. (B) Recurrence-free survival status of stage I CC patients. (C) Heatmap of 8 immune gene expression profiles of stage I CC patients. [file 6699131.f1.zip › 6699131.f1/Figure S1.pdf]

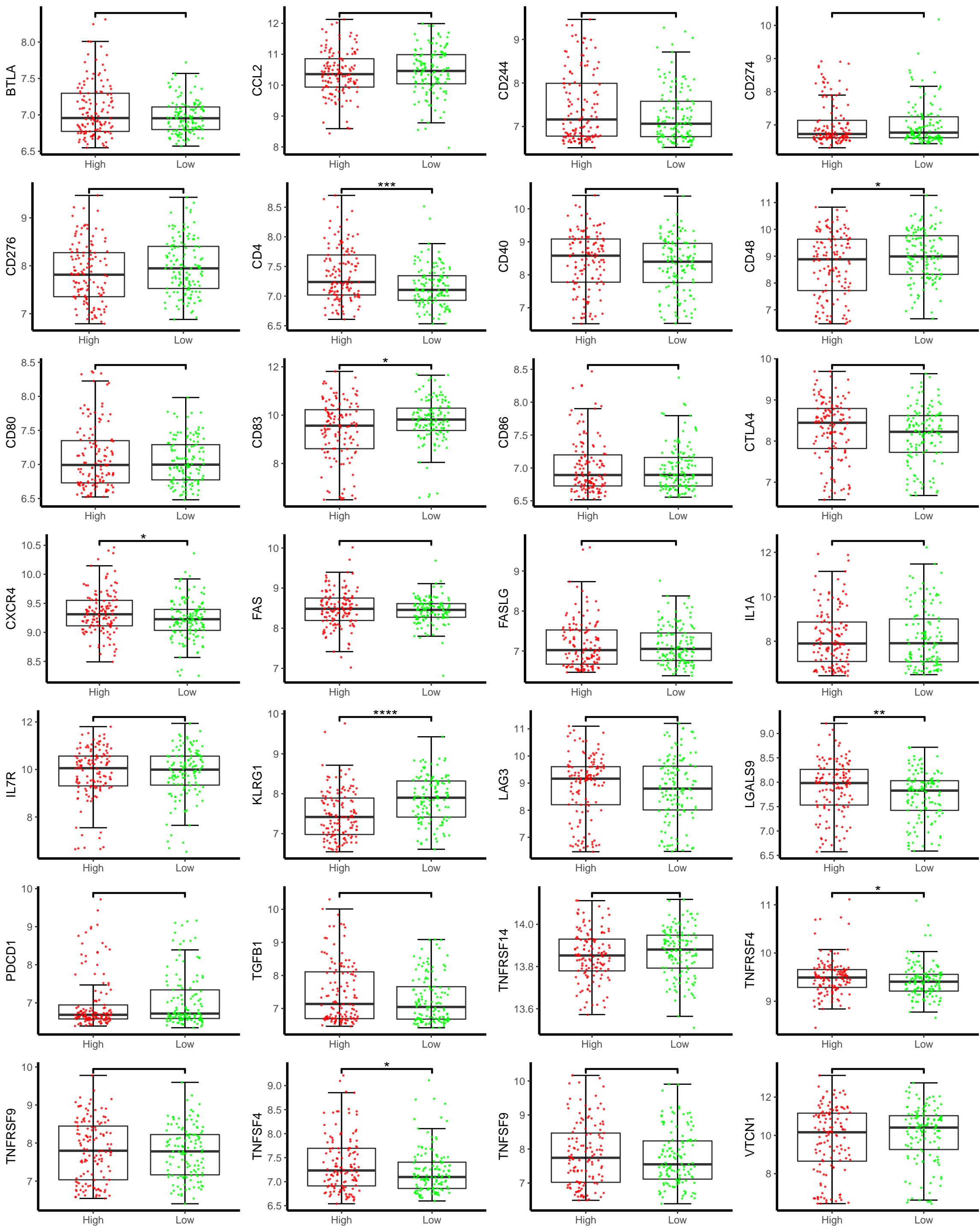

Supplement: Supplementary Materials — Table S1: the detailed clinical information of the samples enrolled in the present study. Table S2: all primer sequences used in this study. Table S3: hazard ratios and 95% CIs and p values of 1811 immune genes via univariate Cox regression analysis. Table S4: the enriched pathways related to the immune risk score. Figure S1: boxplots of 8 immune gene expression values against the risk group in the TCGA dataset. Figure S2: 28 immune checkpoints from the TCGA database were explored between the two risk groups. Figure S3: tumor mutation burden (TMB) from the TCGA database was explored between the two risk groups. Figure S4: the expression of the immune genes in CC tissues was assessed by qRT-PCR and IHC analysis. Figure S5: immune gene risk score analysis of 153 stage I CC samples in the TCGA database. (A) Metabolic gene risk score distribution against the rank of risk score. Median risk score was adopted as the cutoff point. (B) Recurrence-free survival status of stage I CC patients. (C) Heatmap of 8 immune gene expression profiles of stage I CC patients. [file 6699131.f1.zip › 6699131.f1/Figure S2.pdf]

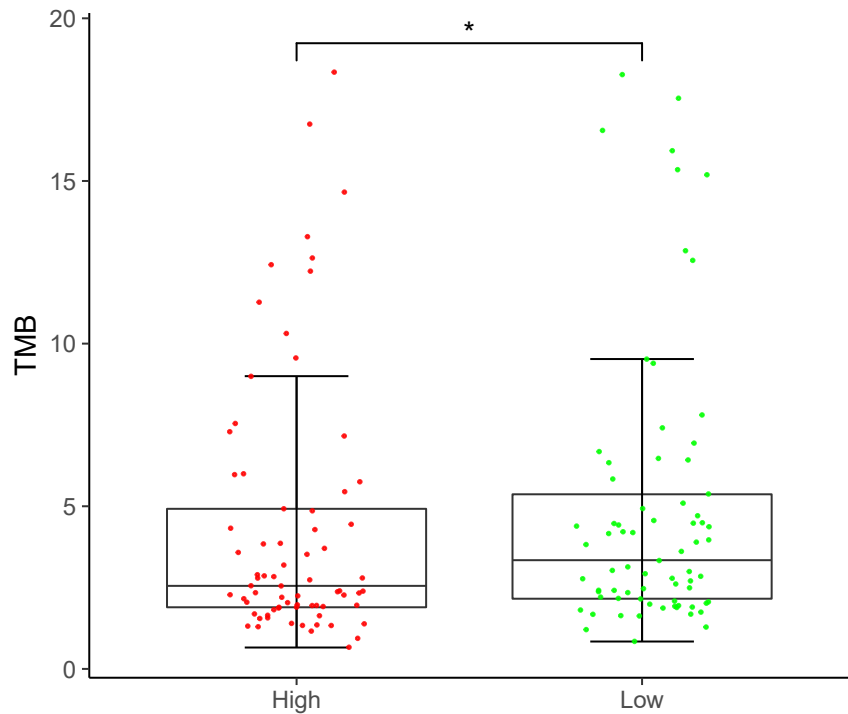

Supplement: Supplementary Materials — Table S1: the detailed clinical information of the samples enrolled in the present study. Table S2: all primer sequences used in this study. Table S3: hazard ratios and 95% CIs and p values of 1811 immune genes via univariate Cox regression analysis. Table S4: the enriched pathways related to the immune risk score. Figure S1: boxplots of 8 immune gene expression values against the risk group in the TCGA dataset. Figure S2: 28 immune checkpoints from the TCGA database were explored between the two risk groups. Figure S3: tumor mutation burden (TMB) from the TCGA database was explored between the two risk groups. Figure S4: the expression of the immune genes in CC tissues was assessed by qRT-PCR and IHC analysis. Figure S5: immune gene risk score analysis of 153 stage I CC samples in the TCGA database. (A) Metabolic gene risk score distribution against the rank of risk score. Median risk score was adopted as the cutoff point. (B) Recurrence-free survival status of stage I CC patients. (C) Heatmap of 8 immune gene expression profiles of stage I CC patients. [file 6699131.f1.zip › 6699131.f1/Figure S3.pdf]

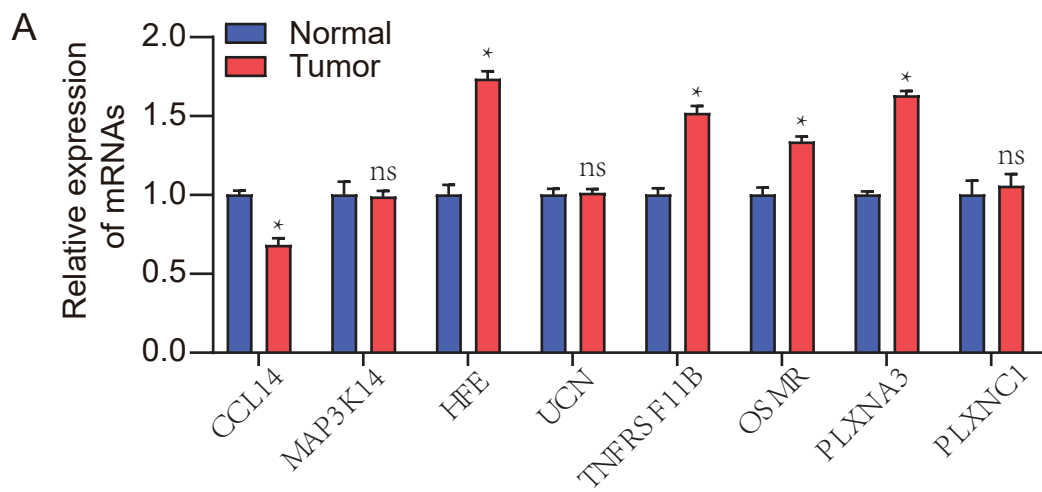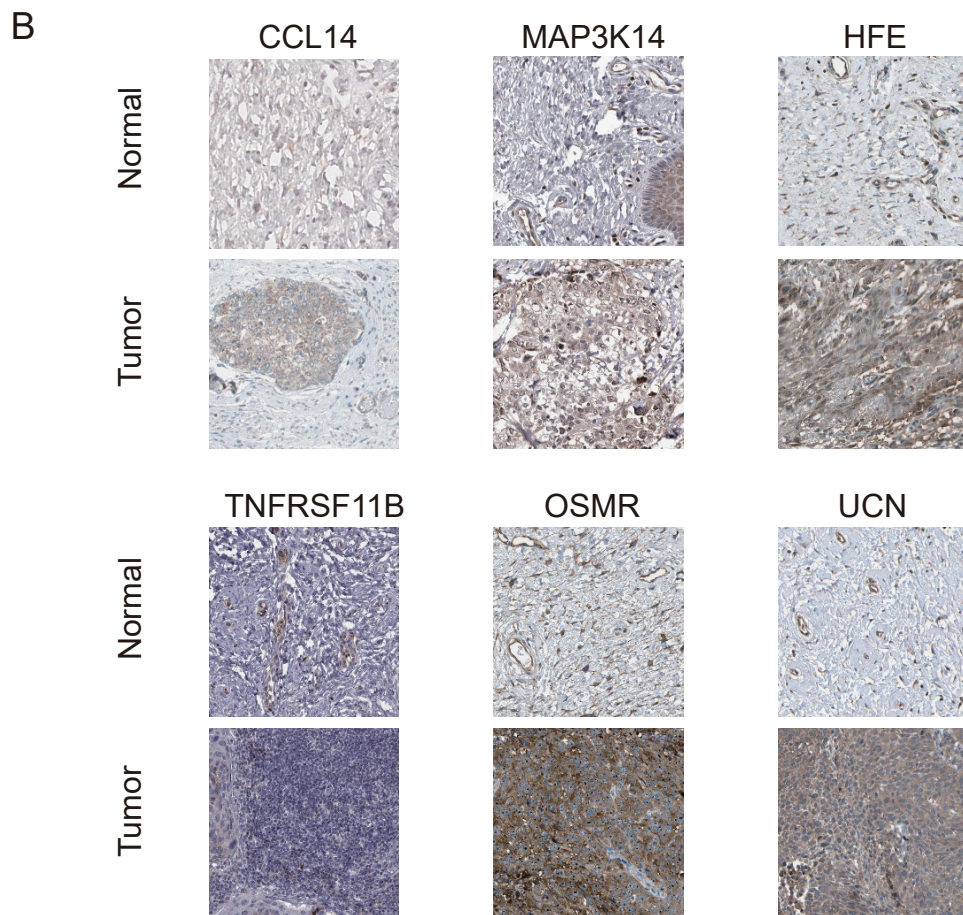

Supplement: Supplementary Materials — Table S1: the detailed clinical information of the samples enrolled in the present study. Table S2: all primer sequences used in this study. Table S3: hazard ratios and 95% CIs and p values of 1811 immune genes via univariate Cox regression analysis. Table S4: the enriched pathways related to the immune risk score. Figure S1: boxplots of 8 immune gene expression values against the risk group in the TCGA dataset. Figure S2: 28 immune checkpoints from the TCGA database were explored between the two risk groups. Figure S3: tumor mutation burden (TMB) from the TCGA database was explored between the two risk groups. Figure S4: the expression of the immune genes in CC tissues was assessed by qRT-PCR and IHC analysis. Figure S5: immune gene risk score analysis of 153 stage I CC samples in the TCGA database. (A) Metabolic gene risk score distribution against the rank of risk score. Median risk score was adopted as the cutoff point. (B) Recurrence-free survival status of stage I CC patients. (C) Heatmap of 8 immune gene expression profiles of stage I CC patients. [file 6699131.f1.zip › 6699131.f1/Figure S4.pdf]

A

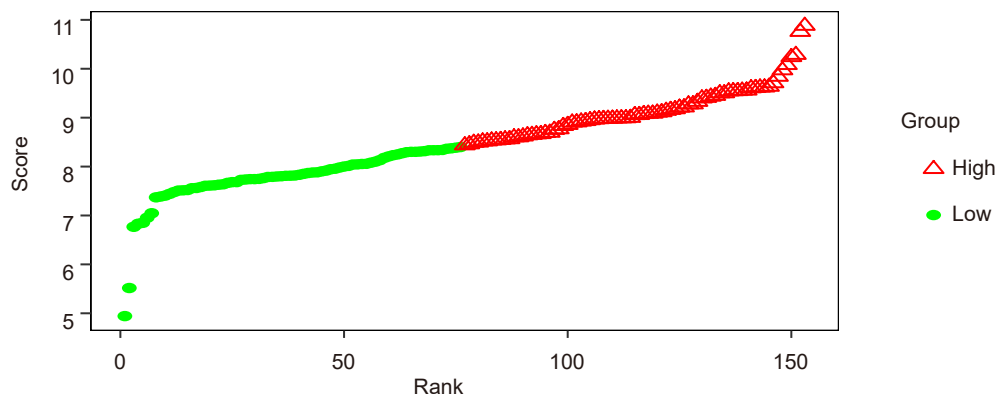

B

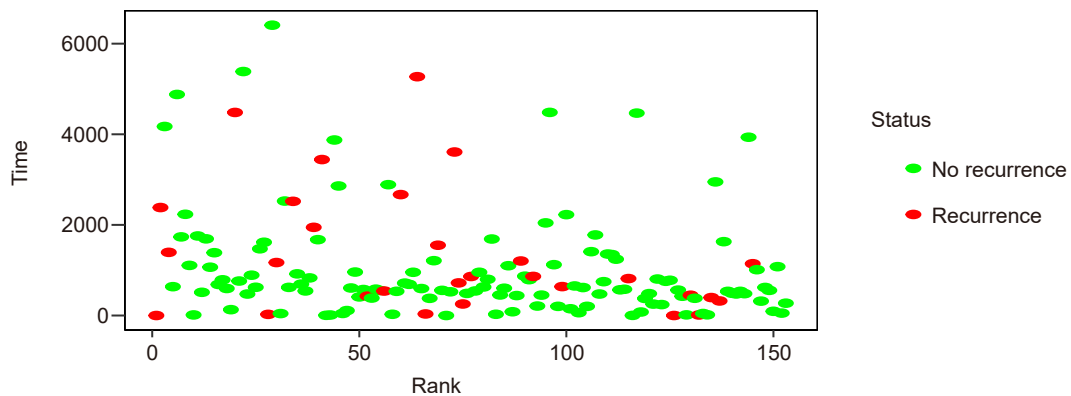

C

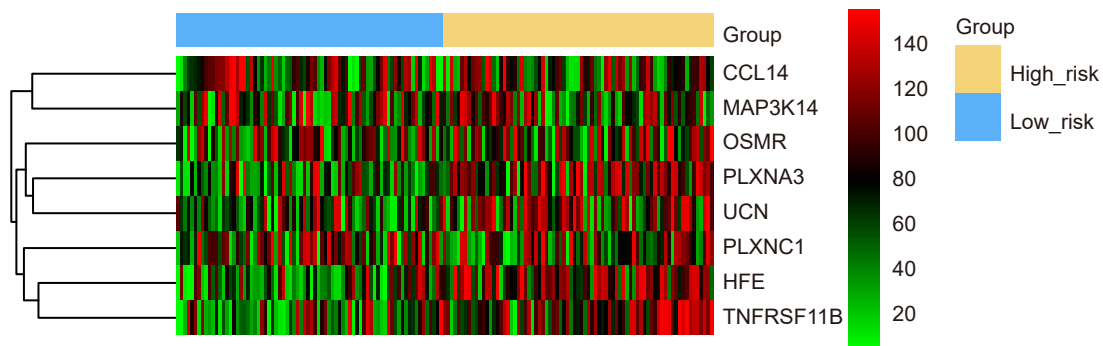

Supplement: Supplementary Materials — Table S1: the detailed clinical information of the samples enrolled in the present study. Table S2: all primer sequences used in this study. Table S3: hazard ratios and 95% CIs and p values of 1811 immune genes via univariate Cox regression analysis. Table S4: the enriched pathways related to the immune risk score. Figure S1: boxplots of 8 immune gene expression values against the risk group in the TCGA dataset. Figure S2: 28 immune checkpoints from the TCGA database were explored between the two risk groups. Figure S3: tumor mutation burden (TMB) from the TCGA database was explored between the two risk groups. Figure S4: the expression of the immune genes in CC tissues was assessed by qRT-PCR and IHC analysis. Figure S5: immune gene risk score analysis of 153 stage I CC samples in the TCGA database. (A) Metabolic gene risk score distribution against the rank of risk score. Median risk score was adopted as the cutoff point. (B) Recurrence-free survival status of stage I CC patients. (C) Heatmap of 8 immune gene expression profiles of stage I CC patients. [file 6699131.f1.zip › 6699131.f1/Figure S5.pdf]
